# Thermodynamically Stable Intermediate in the Course of Hydrogen Ordering from Ice V to Ice XIII

**DOI:** 10.1021/acs.jpclett.3c03411

**Published:** 2024-01-25

**Authors:** Keishiro Yamashita, Thomas Loerting

**Affiliations:** Institute of Physical Chemistry, University of Innsbruck, Innrain 52c, 6020 Innsbruck, Austria

## Abstract

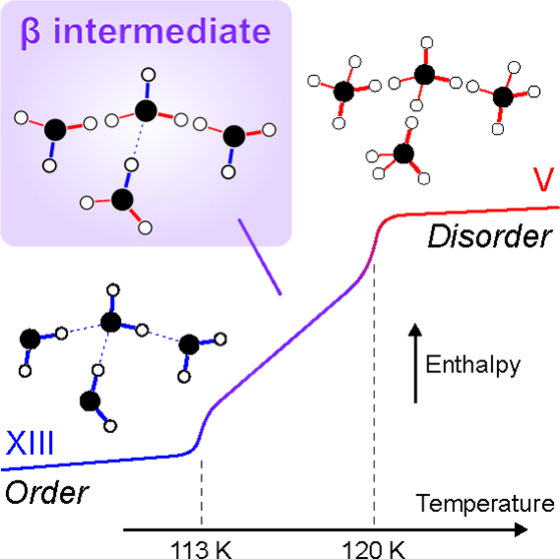

Even though many partially ordered ices are known, it remains elusive to understand and categorize them. In this study, we study the ordering from ice V to XIII using calorimetry at ambient pressure and discover that the transition takes place via an intermediate that is thermodynamically stable at 113–120 K. Our isothermal ordering approach allows us to highlight the distinction of this intermediate from ice V and XIII, where there are clear differences both in terms of enthalpy and ordering kinetics. We suggest that the approach developed in the present work can also reveal the nature of partially ordered forms in the hydrogen order–disorder series of other ice phases.

Water is a simple molecule, but its unique physicochemical properties are key to life as we know it, to many geological processes on and within Earth, and to the evolution of planetary systems. Its crystalline forms, ices, are known for their tremendous structural variety, where 20 polymorphs are experimentally accessible today.^1−3^ Most of the ice polymorphs can be classified into two categories by the orientational alignment of water molecules: hydrogen-disordered phases, in which water molecules orient randomly, and hydrogen-ordered phases, in which water molecules orient in a specific manner.^1−3^ In most cases, each hydrogen-disordered phase has a single ordered counterpart with a symmetrically equivalent oxygen lattice.

In general, ordered structures can be found at lower temperatures for their lower enthalpy, which leads to their lower free energy. On the other hand, disordered structures become dominant at higher temperatures because the contribution of the configurational entropy overcomes the enthalpic disadvantage. Under the prerequisite that the transition is reversible and the system is in equilibrium at isobaric condition, the enthalpy difference (Δ*H*) between the hydrogen order–disorder pair can be related to their difference in configurational entropy (Δ*S*_conf_) which corresponds to the difference in the degree of hydrogen (dis)order through Δ*S*_conf_ = Δ*H*/*T*_c_, where *T*_c_ indicates the equilibrium order–disorder temperature.

The description as a pair of one with full disorder and a counterpart with complete order is an ideal classification. Experimentally observed ice phases are sometimes neither of them, but take partially hydrogen-(dis)ordered structures (e.g., ices III/IX,^4−7^ V,^7,8^ XIV,^9,10^ XV,^11,12^ and XIX^13,14^), in which the molecular orientations feature both some randomness and certain order at the same time. Their understanding is hampered mostly because of kinetic reasons: molecular reorientations become too slow at low temperatures and are often frozen before the ordering process has completed, producing what might be called a glassy state of molecular orientations in which the oxygen arrangement displays long-range order. By contrast, amorphous ices feature a glassy nature of the oxygen atom arrangements. The kinetically frozen, orientational glassy state is often not clearly distinguished from truly thermodynamically stable partially ordered phases (e.g., see refs (12 and 15−18)). Previous experimental studies mostly focused on obtaining samples ordered enough to distinguish them from disordered phases experimentally, such as by calorimetry,^19^ Raman,^20^ and neutron diffraction.^9^ Various approaches have been attempted to enhance the degree of hydrogen order such as acid/base dopant,^9,21^ H/D dopant,^13^ slow cooling,^10^ and cryo-storage.^22^ Nevertheless, resultant “ordered” phases still contain substantial degrees of disorder (ices IX,^4−6^ XI,^21,23−26^ XIV,^9,10^ XV,^11,12^ and XIX^13,14^). It has remained unclear whether such ices represent merely transient states on the way to the ideal ordered ices or whether these ices are thermodynamically stable forms distinct from the ideal ordered ices. Such ambiguity is also shared for some disordered phases such as ices III^5−7^ and V.^7,8^ In other words, the fundamental details of the order–disorder phenomena are still far from complete, despite decades of research on ordered ices.

In this study, we investigate the calorimetric behavior of the ice V–XIII pair as a model case to elucidate the hydrogen ordering process in ice. Here, ice V–XIII is selected for its three properties: (i) a completely ordered configuration can be defined for ice XIII,^9,27,28^ (ii) the hydrogen order–disorder transition takes place reversibly at ambient pressure,^9,19,28^ and (iii) the orientational glass-transition temperature is below the hydrogen order–disorder transition boundary,^19,29^ which means that the water molecules have enough mobility to rearrange the configurations.

Ice V is a thermodynamically stable crystalline phase at around 0.5 GPa and 250 K.^30^ Ice V is recognized as a disordered phase but is also known to contain partial order.^7,8^ In contrast to some other “ordered” phases, its ordered counterpart, ice XIII, can form experimentally in an almost completely ordered structure except for a small degree of remnant disorder.^9,28^ The enthalpic preference of the ice XIII configuration is also confirmed by calculation from density functional theory.^27^ This means that the ideal ordered structure without residual configurational entropy can be defined explicitly for ice XIII. Here, we refer to “ice XIII” as the ideally ordered phase but also transient states that continuously transform into the ideal order.

In practice, pure ice V (without the acid dopant) does not order to produce ice XIII,^31−33^ and the hydrogen ordering needs the assistance of acid dopants like HCl.^19,28^ The acid dopant facilitates hydrogen ordering by the addition of Bjerrum and ionic defects in the crystal lattice.^9,19,28^ Dielectric spectroscopy reveals forty thousand times faster hydrogen dynamics in doped ice V.^29^

The ice V–XIII phase transition takes place reversibly at 110–125 K and ambient pressure,^9,19^ below the temperature of its irreversible decomposition into stacking-disordered ice I_sd._^32^ Moreover, previous studies indicate that the molecular kinetics is unfrozen in HCl-doped ice V/XIII above 103 K (dielectric spectroscopy^29^) or 105 K (calorimetry^19^). The reversibility of the transition and the facile molecular reorientations provide us with direct access to the thermodynamic properties under equilibrium conditions. It should be noted that in the energy landscape at ambient pressure, ice V/XIII is in the metastable basin mostly defined by oxygen sublattice compared to ordinary ice I. Hydrogen order–disorder takes place among the many shallow energy minima within this metastable basin.

Ice V undergoes two-stepped hydrogen-(dis)ordering events upon heating/cooling.^15,19,34^ Authors of previous detailed structural studies put forward the idea that these features are related to separate processes at two different types of hydrogen bonds.^9,15,19,28^ Such an interpretation fits with experimental observations such as the crystal structure model refined from neutron diffraction. However, this idea is too simplified to describe the complicated hydrogen ordering phenomena. Rather than that, other approaches such as a statistical description based on mixtures of configurations would be needed (e.g., see refs (12, 27, and 35)). Thus, a comprehensive understanding of the two-step ordering events is also far from completion.

A slow cooling technique is a common approach to increase the degree of order (e.g., see refs (19 and 28)), but several possible types of ordering processes take place over a temperature range and we can never know whether the product is sufficiently equilibrated, representing a thermodynamically stable phase or just an orientational glassy state frozen transiently. Here, an isothermal annealing approach is introduced that allows us to extract kinetic and thermodynamic properties simultaneously, allowing us to separate and isolate different types of ordering processes. This supersedes the previous state-of-the-art technique of continuous cooling/heating cycles and, thereby, opens the door to elucidate the (dis)ordering process in ice V/XIII in an unprecedented way. This approach provides us with access to the intermediate-ordered ice that has been inaccessible previously.

Figure 1 shows calorimetry scans representing the thermal behavior of ice V/XIII upon cooling at 2 K min^–1^ at ambient pressure. Curve 1 features two exothermic processes upon continuous cooling, corresponding to the two-step hydrogen ordering, as reported.^15,19,34^ To exclude the possibility that the ordering is a single process that takes place with two types of kinetics, e.g., kinetics of bulk and surface, a separate experiment was done. This experiment involves two cooling scans from 134 to 115 K and from 119 to 93 K. In between, 40 min of isothermal annealing was applied for the sample at 119 K, i.e., below the first endotherm, but above the second one. The first scan (Curve 2-1) before the second exotherm is identical to the continuous cooling (Curve 1). In the second scan starting from 119 K after isothermal annealing, ice V/XIII still exhibits the second exotherm at ≈114 K upon cooling (Curve 2-2). This observation rules out the scenario of an orientational glassy state. If the second exothermic feature comes from a heat capacity undershoot in the glassy scenario,^18^ this undershoot should disappear or diminish after providing the system enough time for the molecular reorientations.^29^ Nevertheless, in trace 2-2 in Figure 1, it clearly remains even after a 40 min anneal. Considering the reversibility of the ordering transitions,^19^ these processes are attributed to hydrogen ordering in ice V/XIII. Furthermore, the separated exothermic peaks imply the ordered state of ice V/XIII in this intermediate temperature range has discrepancies in enthalpy from both ices V and XIII. That is, ice V transforms first into an intermediate, and then the intermediate transforms into ice XIII.

**Figure 1:**
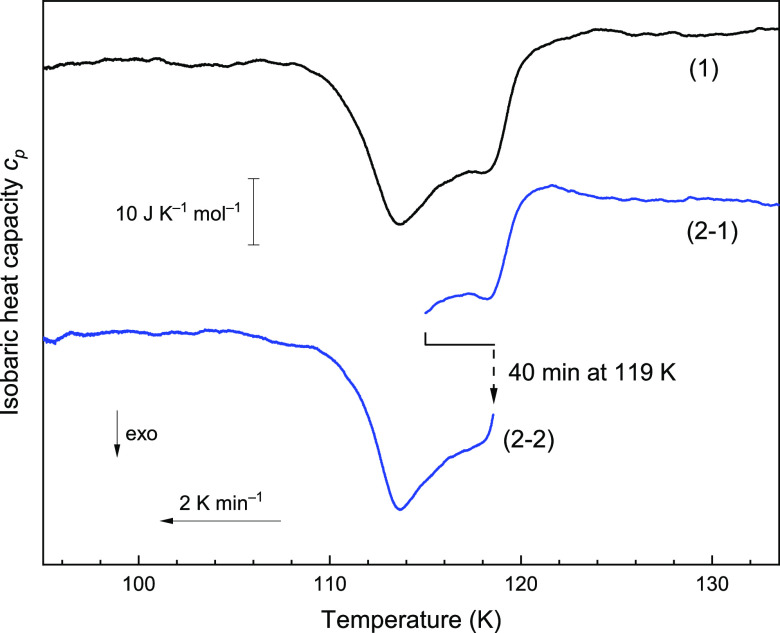
Representative DSC curves of 0.01 M HCl-doped ice V/XIII upon cooling at 2 K min^–1^. (1) Continuous cooling from 134 to 93 K. (2-1) Cooling from 134 to 115 K followed by 40 min annealing at 119 K and (2-2) cooling from 119 to 93 K. Thermograms are aligned with offset by subtracting linear baseline derived for *T* = 125–132 K (curves 1 and 2-1) or 98–104 K (curve 2-2) for clear comparison of the exothermic features.

The next question is whether the intermediate is just a transient state or a thermodynamically stable state as an outcome of equilibration. Figure 2 shows the results of the heating scans for isothermally annealed ice V/XIII at different anneal temperatures *T*_anneal_ = 100–119 K for various anneal times *t*_anneal_ = 0.1–362 min. As seen in the inset of Figure 2, the size of the endotherm increases with annealing time at 110 K. This indicates that hydrogen ordering proceeds with time. In the limit of infinite time, this annealing produces a thermodynamically stable state at *T*_anneal_. At finite times, transient states are encountered that slowly converge to equilibrium. As a result, Δ*H* monotonically increases with *t*_anneal_ and reaches a plateau after long *t*_anneal._ The mere observation of the plateau suggests that the ordering converges to a thermodynamically stable, equilibrated state. In the range of 100–110 K, the same kind of plateau is reached, suggesting that the same type of equilibrated and ordered state is reached. Yet, at 115–119 K, lower-lying plateaus are reached (Figure 2), which suggests that different types of order form in equilibrium.

**Figure 2:**
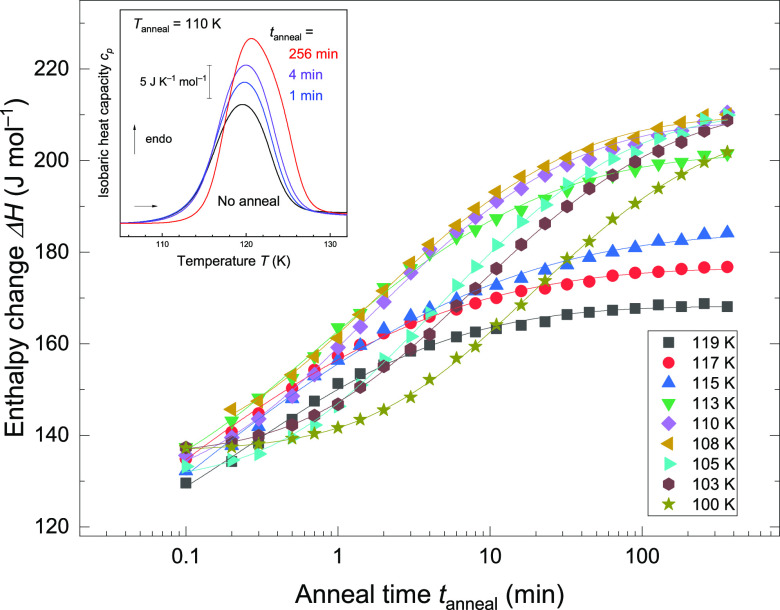
Enthalpy change Δ*H* upon disordering against anneal time *t*_anneal_. The colors and shapes of the symbols correspond to the anneal temperature *T*_anneal_ described in the legend. Solid lines are fitted curves, using eq 1. The inset shows representative thermograms for *T*_anneal_ = 110 K. Representative thermograms are aligned by subtracting the linear baseline derived for *T* = 100–104 K for a clear comparison of the endothermic features.

Here, an exponential-based function is introduced to analyze the development of hydrogen order, as represented by Δ*H*, which converges to a specific value. The actual formula is similar to the modified Johnson–Mehl–Avrami–Kolmogorov (JMAK) equation which is widely used for nucleation and growth^36−39^ as well as kinetic study for hydrogen (dis)ordering in ice VI–XV–XIX.^40^ The Δ*H* increase upon isothermal ordering as a function of time (*t*) can be formulated as
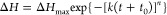
1where *k* is the rate constant and *n* corresponds to the Avrami exponent. Here, Δ*H*_max_ is the maximum enthalpy change that is reached asymptotically after a long *t*_anneal_, and *t*_0_ is the time offset for ordering before isothermal annealing. That is, Δ*H*_max_ represents the limit of infinite time, corresponding to a thermodynamic property. On the other hand, *k* describes the kinetic property, i.e., how fast ordering proceeds toward the equilibrium. In many cases, the value of *n* allows for mechanistic interpretations, but practical interpretations of experimentally derived *n* values for complicated events are not straightforward (e.g., see ref (41)). Thus, we focus on only two properties to extract the characteristics of the hydrogen-ordering behavior of ice V/XIII: (i) thermodynamic (Δ*H*_max_) and (ii) kinetic (*k*).

The thermodynamic property (Δ*H*_max_) is almost constant at 210 J mol^–1^ up to 110 K and then has a clear kink at 110–113 K (Figure 3a), identical to the disordering onset temperature *T*_c_ = 112–113 K of ice XIII.^19,29^ The similar Δ*H*_max_ at *T*_anneal_ = 100–110 K indicates that we deal with one ordered phase in this temperature range, namely ice XIII. At 112–113 K and below, ice XIII appears as the dominant phase, with tiny contaminants of other configurations.

**Figure 3:**
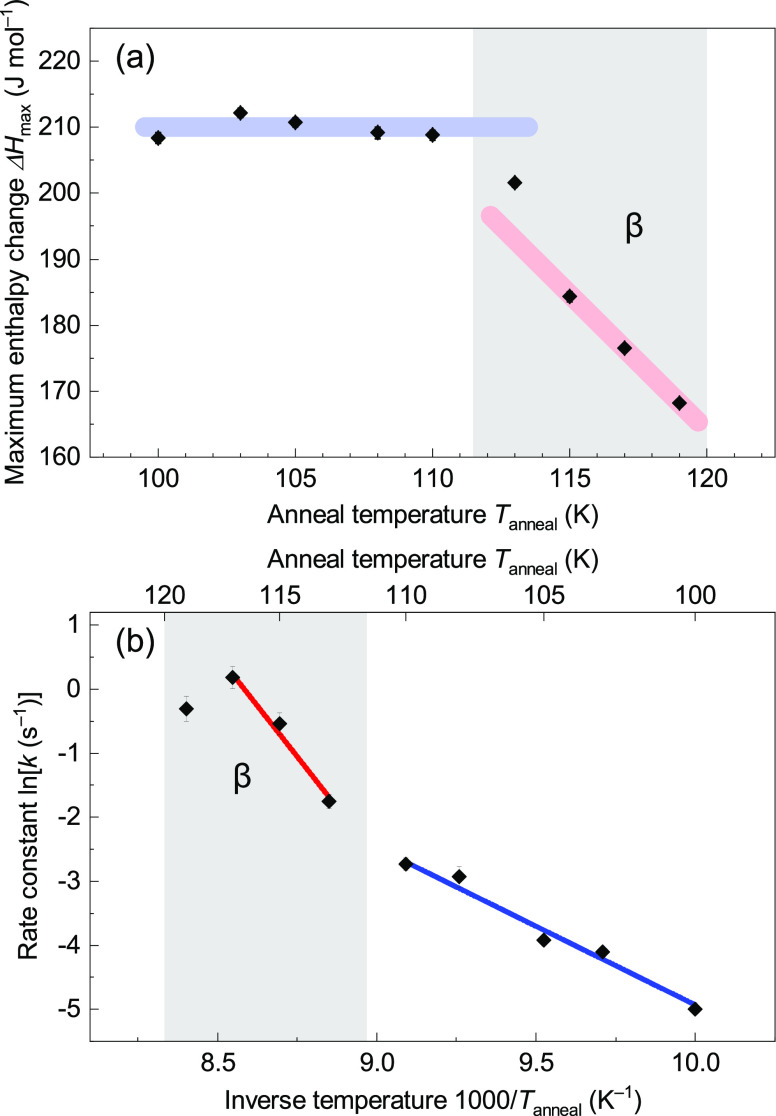
(a) Maximum enthalpy change Δ*H*_max_ and (b) rate constant *k* as fitted parameters for Δ*H* with eq 1 against *t*_anneal_. The thick blue and red lines in (a) are shown as guides to the eye. The blue and red lines in (b) correspond to the linear regressions for *T*_anneal_ = 100–110 K and 113–117 K, respectively.

On the other hand, Δ*H* drastically drops above 113 K. This implies that ice XIII is no longer the dominant phase. Instead, a different type of order starts to dominate. In other words, this threshold of 110–113 K is the crossover temperature between ice XIII and another thermodynamically stable intermediate featuring partial-order, hereafter called the β intermediate of ice V/XIII. The boundary between the β intermediate and ice V is at ≈120 K (SI Figure S2). In other words, the ice V/XIII system features ice V at *T* > 120 K, ice XIII at *T* < 113 K, and the β intermediate at 113–120 K. These correspond to distinct potential minima in the Gibbs free energy landscape into which the system equilibrates.

For the time being, detailed structural characterizations are not available for this β intermediate. However, we can see a hint of the structural discrepancy in the temperature dependence of the *c*-length which drops at 112–120 K assigned for the β intermediate (SI Figure S7; Figure 3 in reference (28)). This is in contrast to the continuous changes in the *a*- and *b*-lengths (Figure 3 in reference (28)). The anisotropy in lattice parameters often reflects the difference in hydrogen ordering manner as seen in the case of ice XV and XIX.^13,14^ The anomaly in *c*-length can be a result of the difference in the hydrogen ordering manner of the β intermediate from both ice V and XIII.

The kinetic property (*k*) increases in general with temperature (Figure 3b) except for *T*_anneal_ = 119 K, close to the upper limit for the β intermediate. If the hydrogen ordering is governed by a single type of kinetics, the rate constant can be described with the pre-exponential factor (*k*_0_) and the activation energy (*E*_a_) as
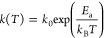
2Two distinct trends are found in the Arrhenius plot (Figure 3b) with the temperature threshold of 110–113 K (see fits in Figure 3b), just like for Δ*H*. The linear fits correspond to activation energies of 20.4 (18) kJ mol^–1^ below 110 K (for ice XIII) and 53 (7) kJ mol^–1^ between 113–117 K (for the β intermediate). That is, there is not only the distinction in Δ*H*_max_, but there are also two types of potential barriers differing in height. This again demonstrates that the β intermediate is clearly different from ice XIII.

Let us now switch from the ordering upon cooling to the disordering upon heating. The kinetics of disordering can be deduced from the shape of the endotherm, especially the peak width and the peak top temperature. At a fixed heating rate, these reflect how fast disordering takes place: narrow peaks and early peak tops reflect fast disordering kinetics. In some cases, a wider peak can arise from two overlapping events (e.g., see ref (42)). Figure 4 summarizes the endotherm shift (Δ*T*_top_) defined as the difference of the peak top compared to the reference case (peak top at *T*_top_ = 119–120 K), which is ice V/XIII cooled at 30 K min^–1^ without annealing in the same calorimetry run.

**Figure 4:**
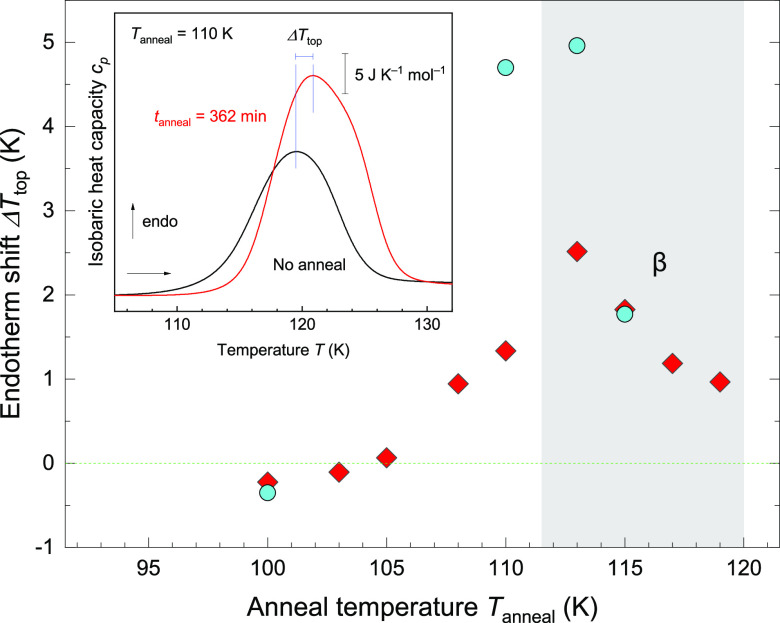
Disordering kinetics inferred from the endotherm shift Δ*T*_top_ against *T*_anneal_ upon heating of ice V/XIII annealed during cooling in the calorimeter. Red diamonds and cyan circles correspond to *t*_anneal_ = 363 and 1000 min, respectively. Inset shows the graphical definition of Δ*T*_top_ for representative thermograms of ice V/XIII annealed at 110 K measured at 30 K min^–1^ in a single DSC run. Representative thermograms are aligned by subtracting linear baseline derived for *T* = 100–104 K for a clear comparison of the endothermic features.

In general, well-ordered structures can survive up to higher temperatures because molecular reorientations are locked by a high energy barrier.^29^ This high barrier originates from the high cost of introducing a defect in the highly ordered structures, as seen in the monotonic increase of Δ*T*_top_ up to *t*_anneal_ ≈ 10 min (SI Figures S3). For *T*_anneal_ = 115–120 K (assigned to the β intermediate), Δ*T*_top_ reaches a plateau of 1–2 K. The plateau Δ*T*_top_ decreases at higher *T*_anneal_, which is simply attributed to a lower degree in hydrogen order as represented by Δ*H*_max_ (see Figure 3a).

The highest Δ*T*_top_ and hence most thermally stable types of hydrogen order are reached for *T*_anneal_ = 113 K (Figure 4). Especially after long anneals of *t*_anneal_ = 1000 min, Δ*T*_top_ reaches up to Δ*T*_top_ ≈ 5 K for *T*_anneal_ = 108–113 K. Such upshifts represent the highest kinetic thermal stability, corresponding to the well-ordered structures of ice XIII with fewer disordered defects. This trend is consistent with the high Δ*H*_max_ values (Figure 3a). In more detail, the large Δ*T*_top_ is not a result of a simple shift of the whole endotherm but an enhancement of a higher-temperature feature seen as a shoulder at ≈125 K (red curve in the inset of Figure 4; see also SI Figure S5). Such two features in the endotherm can be observed in the slow heating of ice XIII^15,19,34^ without the annealing protocol, but the lower-temperature feature is more prominent (detailed in SI Section S5). Here, this higher-temperature feature is assigned mainly to the disordering of well-ordered XIII. That is, the isothermal annealing protocol can produce a properly ordered ice XIII.

Such a well-ordered XIII is expected for all *T*_anneal_ below 113 K from the trend of Δ*H*_max_ (Figure 3). However, Δ*T*_top_ becomes negative after long anneal for *T*_anneal_ below 103 K, despite the high Δ*H*_max_. This instability can be ascribed to tiny disordered domains remaining in the structure from ice V. These cannot convert to the ideally ordered ice XIII structure for kinetic reasons, which makes the overall ordered state the orientational glass. This temperature window of kinetic freezing is consistent with the previous indications from dielectric spectroscopy^29^ and calorimetry.^19^ These glassy remnants behave as orientational defects of the ordered structure and promote disordering. Considering that the formation of the well-ordered ice XIII needs *t*_anneal_ longer than 6 h at *T*_anneal_ below 113 K (SI Figure S3), the reported ice XIII prepared by slow cooling (0.1–0.2 K min^–1^)^9,28^ is considered to be frozen in a transient state. That would be a reason why a small degree of disorder still remains in ice XIII even at 12 K.

In summary, we have developed an isothermal annealing approach involving calorimetric heating scans that provides access to both the kinetics of hydrogen ordering and the thermodynamic properties of the resulting ice, as well as their stability against the disordering. This approach is applied to the case of hydrogen ordering in the ice V/XIII pair and allows us to identify a thermodynamically stable intermediate state called the β intermediate. We focus on the limit of long times, i.e., equilibrated conditions, at ambient pressure avoiding the common uncertainty of ex-situ experiments whether or not the observed ice is transient or thermodynamically stable. Such distinguishments were hampered in many studies on ice polymorphs, especially those that include irreversible changes, due to several factors such as *pT*-dependency in high-pressure preparation (e.g., see refs (17, 42, and 43)).

In more detail, below 113 K, the single completely ordered configuration, known as ice XIII,^9^ is dominant. This boundary has been regarded as the disordering temperature from calorimetry^19^ and dielectric spectroscopy.^29^ Above 120 K, the disordered state, known as ice V, forms with some partial order.^7,8^ Our study points out the existence of the thermodynamically equilibrated β intermediate which is distinct from both ices XIII and V in enthalpy, implying also differences in hydrogen order. The previously unexplained two events upon cooling observed in calorimetry scans^15,19,34^ can now be attributed to these two types of thermodynamic boundaries from our study. Moreover, long annealing at 110–113 K exhibits the highest kinetic thermal stability of ice XIII against disorder upon heating, which is attributed to a well-ordered structure. This also suggests that the ordering of ice XIII may proceed further when using appropriate annealing protocols, superseding earlier slow-cooling literature protocols.

These findings highlight the complexity of the hydrogen (dis)ordering phenomena, far from the picture of one-by-one pairs of ordered and disordered ice forms. Different types of order can develop, as clarified through the recent discovery of hydrogen sublattice polymorphism.^13,14^ In the present work, we go one step beyond the previous ice XIX study and show that the β intermediate represents a thermodynamically stable state of partial order. Further elaboration will need computational approaches (e.g., see refs (27 and 44)) and their complementation with experimental observations such as vibrational spectroscopy^20^ and neutron diffraction.^9,28^ Such an intermediate can show up in other ice phases or would be dominant ubiquitously. Specifically, the known “ordered” phases which retain substantial degrees of disorder, such as ices IX,^4−6,33^ XI,^21,23−26^ XIV,^9,10^ XV,^11,12^ and XIX^13,14^ may not be the most ordered phases but represent intermediates just like the β intermediate revealed here.

## Experimental methods

Ice V was prepared by crystal–crystal transitions starting from ice I_h_ containing 0.01 M HCl upon isobaric heating of at 0.5 GPa up to ≈250 K using a piston–cylinder cell, following the established procedure.^9,19,45^ Afterward, the samples were quenched at 77 K and retrieved at ambient pressure. The hydrogen (dis)ordering processes of ice V at ambient pressure were investigated by differential scanning calorimetry (DSC). Before all the measurements, the sample was heated once to 134 K to erase any kind of hydrogen order from ice XIII and to produce ice V. This ambient-pressure preprocess eliminates the uncertain factors on the hydrogen order which potentially occur at high pressures during the preparation.

Three sets of DSC runs were performed to focus on (1) the hydrogen ordering process upon cooling, (2) the ordering process as a result of isothermal annealing, and (3) the disordering behaviors of long-annealed samples upon heating. For run set (1), cooling scans were collected at 2 K min^–1^ in *T* = 93–134, 115–134, and 93–119 K as shown in Figure 1. The last scan was measured after isothermal annealing at 119 K for 40 min after the second scan.

For each scan in run set (2), the sample was cooled at 30 K min^–1^ to specific anneal temperatures (*T*_anneal_ = 100–119 K). After annealing for a certain anneal time (*t*_anneal_ = 0.1–362 min), the sample is quenched to 93 K. The thermal response to hydrogen disordering, an endotherm, was measured upon heating to 134 at 30 K min^–1^ (e.g., inset of Figure 2). The thermograms with the same anneal temperature *T*_anneal_ were measured in a single DSC run changing *t*_anneal_. For run set (3), other data for longer *t*_anneal_ values up to 1000 min were also taken in separate DSC runs. The enthalpy change (Δ*H*) upon disordering was evaluated by the integration of the endotherm after background subtraction and normalization. Further details are given in SI Section S1.

## Supporting Information Available

Complementary data and description including (S1) details of experimental procedures for sample preparation and calorimetry, (S2–S5) representative thermograms and plots with different axis settings for ordered forms of ice V/XIII after isothermal annealing protocols, and (S6) temperature dependence of lattice parameter *c* derived from X-ray diffraction (PDF)
