# Supplementary material for: Thermodynamically Stable Intermediate in the Course of Hydrogen Ordering from Ice V to Ice XIII
Source: J Phys Chem Lett. 2024 Jan 25;15(4):1181–7. doi: 10.1021/acs.jpclett.3c03411 (PMC10839903; doi:10.1021/acs.jpclett.3c03411)
Supplement: Supplementary file 1 — jz3c03411_si_001.pdf [file jz3c03411_si_001.pdf]

Supplemental Information

Thermodynamically Stable Intermediate in the Course of Hydrogen Ordering from Ice V to Ice XIII

Keishiro Yamashita*, Thomas Loerting
Institute of Physical Chemistry, University of Innsbruck, Innrain 52c, 6020 Innsbruck, Austria
*e-mail: Keishiro.Yamashita@uibk.ac.at

S1 Details of experimental procedures

Sample preparation

0.01 M HCl solution was poured into an indium container precooled at liquid nitrogen temperature. The sample was pressurized under liquid nitrogen temperature, using a high-pressure piston cylinder with an 8 mm bore together with a commercial "universal material testing machine" (Zwick, model BZ100/TL3S).[1–3] Ice V finally forms as the thermodynamically stable phase *via* the solid-solid transitions (I → III/IX → II → V)[4,5] upon isobaric heating at 0.5 GPa up to *ca.* 250 K. Afterwards, the samples were quenched to 77 K and retrieved at ambient pressure. The phases of obtained samples were confirmed as ice V by x-ray diffraction (same procedure as described in S6).

Calorimetric measurements

The hydrogen ordering of recovered ice V was investigated under He flow by differential scanning calorimetry (DSC) using DSC8000 (Perkin Elmer). In each DSC scan, the sample was once heated to 134 K and then cooled down by 30 K min^{-1} to specific anneal temperatures (T_{anneal} = 100–119 K). After annealing for a specific anneal time (t_{anneal} = 0.1–362 min), the sample was quenched to 83 K. The hydrogen disordering was measured upon heating to 134 K at 30 K min^{-1}. Such annealing and heating scans were repeated with the same anneal temperature T_{anneal} but changing t_{anneal} in a single DSC run.

Before and after these repetitive schemes, ice V cooled down by 30 K min^{-1} without annealing was also measured to compensate for the temperature offset varying among runs with different T_{anneal} due to the slight difference in the preparation of the measurement such as the amount and the packing of the ice powders. The heating scan data were subtracted by a heat scan of ice I transformed from the sample once heated up to 253 K, followed by normalization using the heat of melting for hexagonal ice I_h at 273 K, namely 6012 J mol^{-1}.[6] The enthalpy change upon disordering was calculated as the peak area after subtraction of the line between the beginning and end temperature. Other data sets with longer t_{anneal} up to 1000 min were also measured to examine the calorimetric trends in the extended time scale.

S2 Endotherms of disordering in ice V/XIII

Figure S1 represents the calorimetric behaviors of ice V/XIII with T_{anneal} = 110 K upon heating at 30 K min^{-1}. The thermogram shows endothermic features centered at ≈120 K. The ice V-XIII pair undergoes reversible hydrogen ordering-disordering transitions at ambient pressure (*e.g.*, ref 7). For ice V/XIII continuously cooled at 30 K min^{-1}, the enthalpy change ΔH upon the hydrogen disordering (green curve in Figure S1) is estimated to be 132 J mol^{-1}. Since the order-disorder transtition of ice V/XIII is reversible at ambient pressure, this value can be converted into the entropy change ΔS_{conf} from $\Delta H = T_c \, \Delta S_{conf}$, with transition temperature T_c where Gibbs free energies of ice V and the ordered state are equal. Here, given T_c = 112 K as the onset temperature same as ref 7, ΔS_{conf} = 1.18 J K^{-1} mol^{-1} corresponding to 35% of Pauling entropy (ΔS_P = 3.37 J K^{-1} mol^{-1})[8] for continuous cooling at 30 K min^{-1}. The degree of ordering for ice V/XIII can be enhanced up to ~66 % by slow cooling.[7,9]

Figure S1 also represents the effect of isothermal annealing of ice V/XIII at T_{anneal} = 110 K, below 120 K, the hydrogen ordering temperature observed upon cooling.[7] The isothermal annealing enhances the endotherm, which corresponds to the increase in the degree of hydrogen ordering. Moreover, the endothermic features also show an upshift toward higher temperatures for longer t_{anneal}, implying kinetically higher thermal stability.

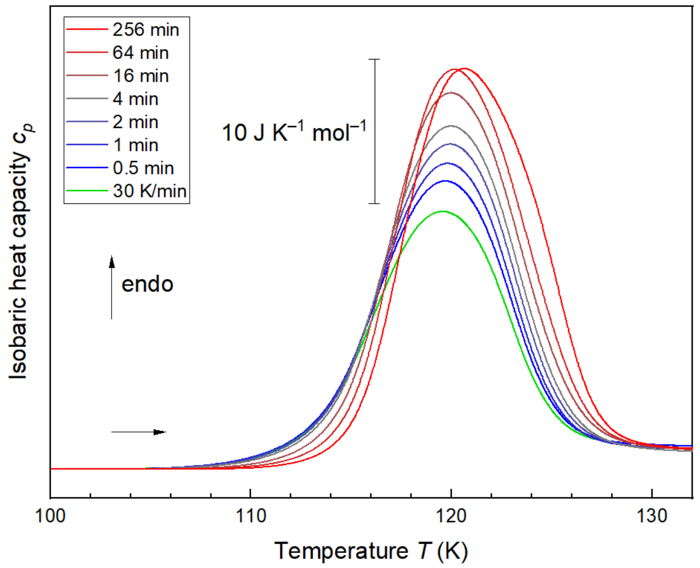

Figure S1 Representative thermograms of ice V/XIII annealed at 110 K for different t_{anneal}. The heating scan for ice V/XIII cooled continuously at 30 K min^{-1} without annealing is described by the green curve for comparison. The thermograms were measured upon heating at 30 K min^{-1} in a single DSC run. All the thermograms were aligned by subtracting linear baseline derived for T = 100–104 K for clarity.

S3 Anneal temperature variation with constant anneal time

Figure 2 summarizes the enthalpy change ΔH and the endotherm shift ΔT_{top} upon disordering of ice V/XIII annealed for 40 min at different T_{anneal}. The thermograms were measured in a single run. According to the T_{anneal} increase, ΔH also monotonically increases up to $T_{anneal} \approx 109$ K but decreases at higher temperatures. After it starts to decrease, ΔH is still higher up to $T_{anneal} \approx 120$ K than the value for $T_{anneal} \approx 93$ K. On the other hand, ΔH discontinuously drops to 123 J mol^{-1}, almost the same as that for ice V/XIII directly quenched from 134 K. This indicates that hydrogen ordering cannot take place at $T_{anneal} > 120$ K and its ordered state is the same as the simple quenching scheme.

Similar to the temperature region where ΔH decreases but remains higher than simple quenching, the endotherm shift ΔT_{top} is also large, similar to those observed in Figure 4 with different t_{anneal} ranges. Moreover, ΔT_{top} for $T_{anneal} > 120$ K is below zero. Combining the idea for this temperature region is the same as the direct quenching scheme: this negative ΔT_{top} is consistent with the scenario, which proposes the remaining disordered matrices in ice V/XIII frozen at low temperatures kinetically decreases the thermal stability of the ordered state (as described in the main text).

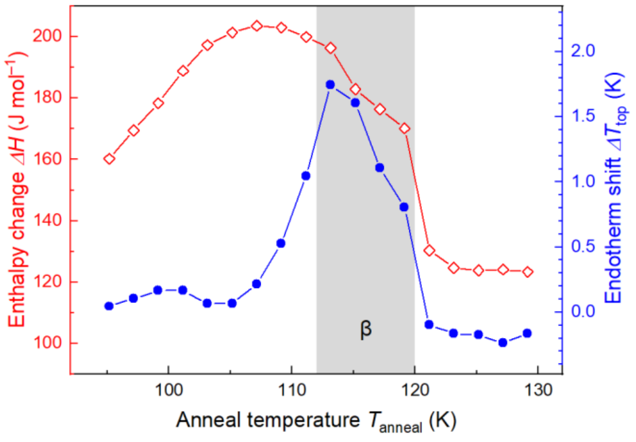

Figure S2 Enthalpy change ΔH (open red diamonds) and endotherm shift ΔT_{top} (filled blue circles) against anneal temperature T_{anneal}. The heating scans were measured at 30 K min^{-1} after annealing procedures at T_{anneal} as described in the main text. The anneal time t_{anneal} was fixed at 40 min. All data points are measured in the same DSC run.

S4 Endotherm shift vs. enthalpy change for the disordering of ice V/XIII

Figure S3 shows that for anneal temperature T_{anneal} above 104 K, endotherm shift ΔT_{top} increases monotonically until short annealing time t_{anneal}, mostly up to 10 min. For T_{anneal} above 115 K, ΔT_{top} reaches almost a plateau after that while T_{anneal} = 108–113 K shows drastic increases of ΔT_{top} at longer annealing. On the other hand, ΔT_{top} scarcely increases for T_{anneal} below 104 K and even decreases at longer t_{anneal}.

Figure S4 shows the relation between ΔT_{top} and enthalpy change ΔH for ice V/XIII. For T_{anneal} > 115 K, ΔT_{top} increases almost linearly against ΔH. For T_{anneal} = 110 and 113 K, the trend is similar to T_{anneal} > 115 K at lower ΔH but ΔT_{top} drastically increases at higher ΔH, finally reaching 5 K. On the other hand, ΔT_{top} remains small or even decreases for T_{anneal} < 103 K. Even though observed ΔH and the ΔH-ΔT_{top} relation slightly vary among the measurements, these comparison trends are reproducible and reveal the absence of systematic direct relation between ΔH and ΔT_{top} over the whole T_{anneal} range.

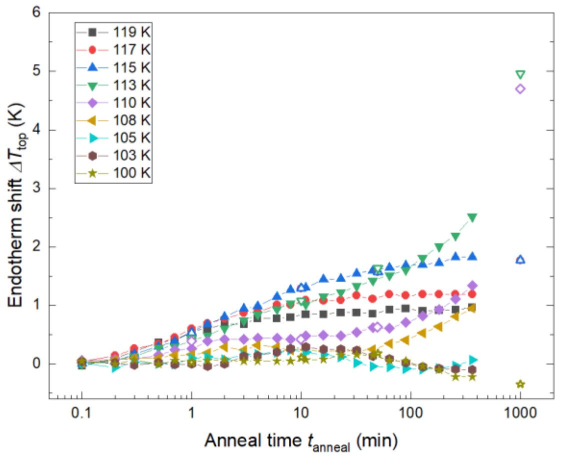

Figure S3 Endotherm shift ΔT_{top} against t_{anneal} upon heating of ice V/XIII annealed during cooling in the calorimeter. The colors and shapes of symbols correspond to the anneal temperature T_{anneal}. Open symbols represent additional data sets from different DSC runs with longer t_{anneal} up to 1,000 min.

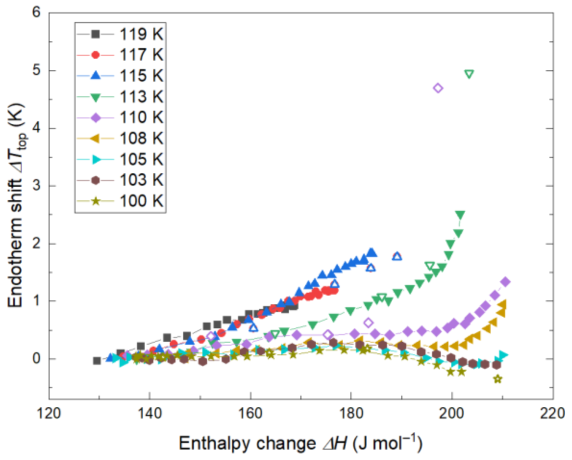

Figure S4 Endotherm shift ΔT_{top} against enthalpy change ΔH upon heating of ice V/XIII annealed during cooling in the calorimeter for the same data set as in Figures 2 and S3. Open symbols represent additional data sets from different DSC runs with longer t_{anneal} up to 1,000 min.

S5 Kinetic thermal stabilities of ordered states with long anneal time

After isothermal annealing with longer t_{anneal} = 1,000 min, the endotherm profiles (Figure S5) represent the disordering behaviors of equilibrated ordered states. Long annealed ice V/XIII with T_{anneal} = 115 K and 100 K respectively shows the slight upshift and downshift of the endotherm compared to continuous cooling at 30 K min^{-1}, as discussed in the main text (Figure 4).

Remarkably, Ice V/XIII annealed at T_{anneal} = 110 and 113 K for 1000 min has a peak top at ≈125 K, corresponding to ΔT_{top} = 5 K (SI Figure S5). In addition, the endotherm also has a shoulder at the lower-temperature side. The kink of this shoulder is located at a temperature similar to the single endotherm with ΔT_{top} ≈1–2 K observed for T_{anneal} = 115–120 K (β intermediate). The existence of the lower-temperature features in the β intermediate and short-annealed XIII implies that the disordering takes place in two processes: one from less-ordered or β intermediate at lower temperature and another one from a more thermally stable ordered form. Thus, the prominent higher-temperature feature is certainly attributed to the well-ordered ice XIII.

It should be noted that these disordering behaviors are affected by kinetic factors. In this sense, the interpretations of the two features are mostly identical to the common explanation as surface and bulk phenomena, eliminated in the case of ordering (Figure 1). That is, less-ordered ice XIII or β intermediate contain orientational defects, corresponding to the surface while well-ordered ice XIII lacks such defects, corresponding to the bulk. The former transform to ice V quickly at lower temperature while the latter survives kinetically at higher temperatures.

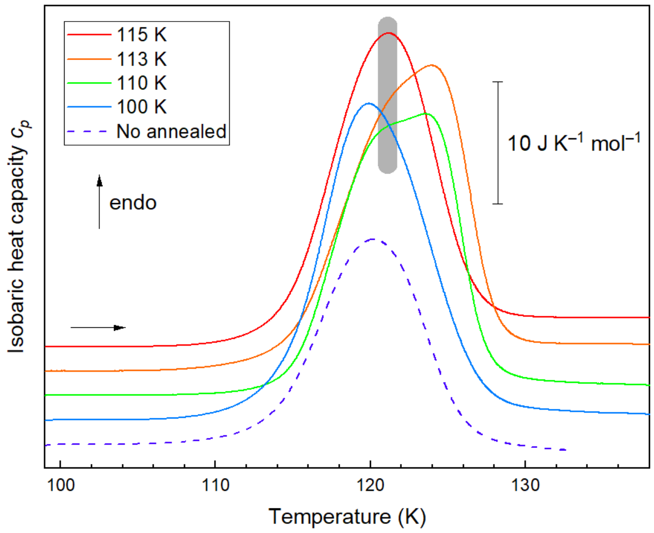

Figure S5 Representative thermograms of 0.01 M HCl-doped ice V/XIII annealed for 1,000 min at T_{anneal}. A thermogram for ice V/XIII continuously cooled at 30 K min^{-1} is also described for comparison. All the data were measured at 30 K min^{-1}. The thick gray vertical line is a guide to the eye for the peak top for T_{anneal} = 115 K and shoulders for T_{anneal} = 110 and 113 K. All the thermograms were aligned with offsets by subtracting linear baseline derived for T = 100–104 K for clarity.

The two distinct features are reminiscent of the endotherms observed upon slow heating of ice V/XIII at ramp rates below 5 K min^{-1} in the reported calorimetric studies.[7,9,10] On the other hand, the higher-temperature features in the previous studies (see Figure 3 in ref 7 or Figure 2 in ref 9) are less prominent than well-ordered XIII (Figure S5). These observations upon the slow heating can be interpreted as the transient ordering in which well-ordered domains of ice XIII develop below 113 K during the heating. The transient ordering is a well-known phenomenon showing an exotherm before the disordering endotherm, especially a case of ice VI/XV in the orientationally glassy state.[9,11–13]

Such interpretation is supported by slow heating scans for annealed ice V/XIII. The thermograms in Figure S6 are almost the same as those in S3 except for the slow heating rate of 10 K min^{-1} instead of 30 K min^{-1}. The overall trends of ΔH and ΔT_{top} are similar to those in Figure S2 except for the magnitude, which is attributed to the transient ordering during the heating.

With 40-min isothermal annealing protocols, the heating scan clearly shows the two features even at 10 K min^{-1} (Figure S6), still much faster than the previous studies (2.5–5 K min^{-1}).[7,9,10] Here, the lower-temperature feature is the most prominent, similar to the reported studies.[9,10] This feature retains its position at ≈117 K up to T_{anneal} = 109 K but shifts to higher temperatures for T_{anneal} = 111–119 K. Above T_{anneal} = 121 K, this lower-temperature feature jumps back to ≈117 K. On the other hand, the higher-temperature feature observed as a shoulder at 121 K remains at the same temperature regardless of T_{anneal}, implying that this disordering event is less dependent on the annealing process than the lower-temperature feature. This suggests that the higher-temperature feature is of an ordered state, where kinetic stability does not vary with thermal history. That is the well-ordered XIII is in an ideally ordered structure without orientational defects. In comparison to this, we assign the lower-temperature features to the disordering from less-ordered ice XIII or the β intermediate containing the orientational defects which promote the disordering.

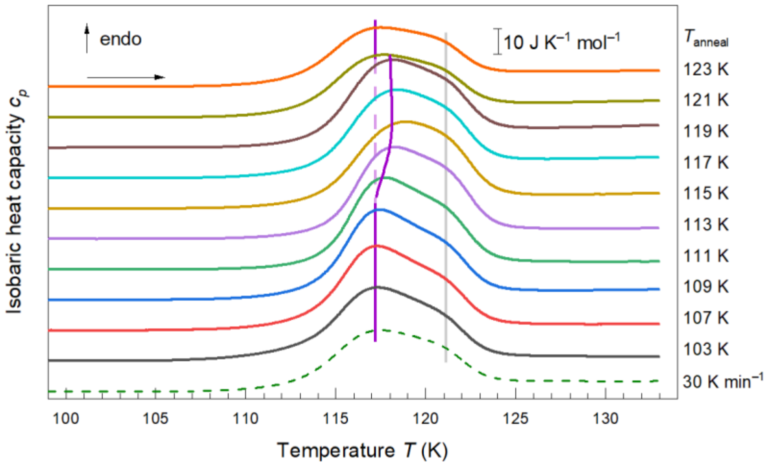

Figure S6 Representative thermograms of 0.01 M HCl-doped ice V/XIII annealed for 40 min at T_{anneal}. A thermogram for ice V/XIII continuously cooled at 30 K min^{-1} is also described for comparison. All the data were measured at 10 K min^{-1}. The violet and grey lines are for the eye guides of the trends of two distinct endothermic features. All the thermograms were aligned with offsets by subtracting linear baseline derived for T = 100–104 K for clarity.

S6 X-ray diffraction

Lattice parameters are known as a key trace of hydrogen ordering.[14–17] From a close look at Figure 3 in literature,[15] we notice that the *c*-length may have a non-monotonic change at 110–120 K, in the range where we identify the intermediate state from calorimetry here. Nevertheless, the data points are sparse and recorded during heating/cooling which would warrant a reinvestigation with closely spaced data points.

We measured x-ray diffraction of ice V/XIII upon hydrogen (dis)ordering using an x-ray diffractometer (D8 Advance, Bruker) with the Bragg-Brentano geometry with Cu*Kα* radiation source. The sample was quickly transferred at liquid nitrogen temperature to a low-temperature chamber (FMB Oxford Ltd.) on the diffractometer. The sample was once heated up to 132 K, then cooled and heated step-by-step between 80 K and 136 K at ≈200 Pa. The diffractograms were recorded by θ-θ scan after 30-min equilibrations. The lattice parameters of ice V/XIII were derived from the Rietveld analysis using the *GSAS* program[18] implemented in the *EXPGUI* software.[19] The *A*2/*a* model of ice V[20] was selected for simple and stable refinement procedures over the whole temperature range. Some tiny Bragg peaks uniquely assignable for $P2_1/a$ model of ice XIII (*e.g.*, $31\bar{2}$ at $2\theta \approx 32.5°$) were observed at low temperatures, but they are not taken into account in the analyses because the lattice parameter definitions are consistent for both models and the intensities of the additional peaks are little in the x-ray diffraction and similar order of the noise of lab-source instrument.

The *c*-length decreases according to the temperature decrease (Figure S7), but its trend is off from the extrapolations from regions above 120 K and below 112 K, assignable for ice V and XIII, respectively. Such a gap was partly observed in the neutron study (See Figure 3 in ref 15). If ice V simply undergoes a simple continuous ordering to ice XIII (*e.g.*, monotonic increase in population of ideal ice XIII structure upon cooling) the temperature dependence will be almost monotonic (as observed for *a* and *b*-lengths used as the characteristic feature of (dis)ordering in refs 14 and 15). The data collection took more than 2 h at each temperature, sufficient to reach equilibrium (see plateau of ΔH in Figure 2), meaning that this observation is in a mostly equilibrated state rather than a transient state. This equilibration is also supported by the little hysteresis between the cooling and heating. This anomaly in *c*-length implies that the β intermediate proposed from the calorimetry may take a specific hydrogen-ordering manner different from both ices V and XIII.

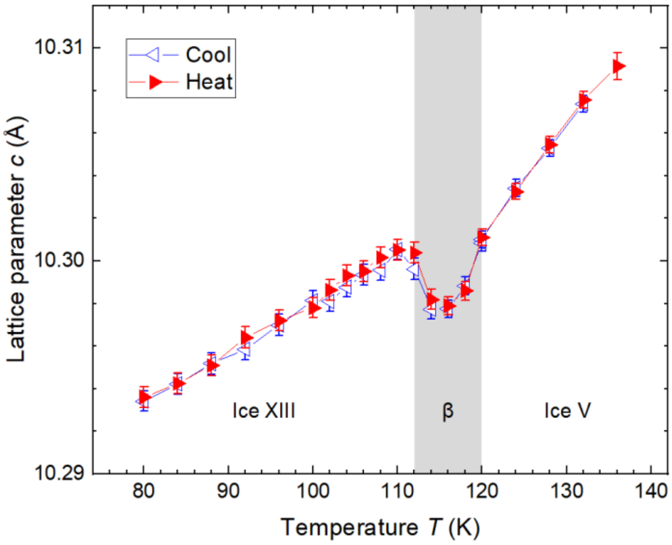

Figure S7 Temperature dependence of lattice parameter *c* of ice V/XIII upon hydrogen (dis)ordering from x-ray diffraction at ≈200 Pa. The lattice parameters and their uncertainties were derived from the Rietveld analysis using the *A*2/*a* model of ice V.

References

(1) Seidl, M.; Amann-Winkel, K.; Handle, P. H.; Zifferer, G.; Loerting, T. From Parallel to Single Crystallization Kinetics in High-Density Amorphous Ice. *Phys. Rev. B - Condens. Matter Mater. Phys.* **2013**, *88* (17), 1–6.

(2) Seidl, M.; Fayter, A.; Stern, J. N.; Zifferer, G.; Loerting, T. Shrinking Water's No Man's Land by Lifting Its Low-Temperature Boundary. *Phys. Rev. B* **2015**, *91* (14), 1–14.

(3) Seidl, M.; Fayter, A.; Stern, J. N.; Amann-winkel, K.; Bauer, M.; Loerting, T. High-Performance Dilatometry under Extreme Conditions. *Proc. 6th Zwick Acad. Day 2015* **2015**.

(4) Whalley, E. Energies of the Phases of Ice at Zero Temperature and Pressure. *J. Chem. Phys.* **1984**, *81* (9), 4087–4092.

(5) Petrenko, V. F.; Whitworth, R. W. *Physics of Ice*; Oxford University Press: Oxford, 2002.

(6) Hallbrucker, A.; Mayer, E. Calorimetric Study of the Vitrified Liquid Water to Cubic Ice Phase Transition. *J. Phys. Chem.* **1987**, *91* (3), 503–505.

(7) Salzmann, C. G.; Radaelli, P. G.; Finney, J. L.; Mayer, E. A Calorimetric Study on the Low Temperature Dynamics of Doped Ice V and Its Reversible Phase Transition to Hydrogen Ordered Ice XIII. *Phys. Chem. Chem. Phys.* **2008**, *10* (41), 6313–6324.

(8) Pauling, L. The Structure and Entropy of Ice and of Other Crystals with Some Randomness of Atomic Arrangement. *J. Am. Chem. Soc.* **1935**, *57* (12), 2680–2684.

(9) Rosu-Finsen, A.; Salzmann, C. G. Benchmarking Acid and Base Dopants with Respect to Enabling the Ice V to XIII and Ice VI to XV Hydrogen-Ordering Phase Transitions. *J. Chem. Phys.* **2018**, *148* (24), 244507.

(10) Sharif, Z. New Insights into Water's Phase Diagram Using Ammonium Fluoride, 2020.

(11) Shephard, J. J.; Salzmann, C. G. The Complex Kinetics of the Ice VI to Ice XV Hydrogen Ordering Phase Transition. *Chem. Phys. Lett.* **2015**, *637*, 63–66.

(12) Rosu-Finsen, A.; Salzmann, C. G. Origin of the Low-Temperature Endotherm of Acid-Doped Ice VI: New Hydrogen-Ordered Phase of Ice or Deep Glassy States? *Chem. Sci.* **2019**, *10* (2), 515–523.

(13) Rosu-Finsen, A.; Amon, A.; Armstrong, J.; Fernandez-Alonso, F.; Salzmann, C. G. Deep-Glassy Ice VI Revealed with a Combination of Neutron Spectroscopy and Diffraction. *J. Phys. Chem. Lett.* **2020**, *11* (3), 1106–1111.

(14) Salzmann, C. G.; Radaelli, P. G.; Hallbrucker, A.; Mayer, E.; Finney, J. L. The Preparation and Structures of Hydrogen Ordered Phases of Ice. *Science (80-.).* **2006**, *311* (5768), 1758–1761.

(15) Salzmann, C. G.; Rosu-Finsen, A.; Sharif, Z.; Radaelli, P. G.; Finney, J. L. Detailed Crystallographic Analysis of the Ice V to Ice XIII Hydrogen-Ordering Phase Transition. *J. Chem. Phys.* **2021**, *154* (13), 134504.

(16) Gasser, T. M.; Thoeny, A. V; Fortes, A. D.; Loerting, T. Structural Characterization of Ice XIX as the Second Polymorph Related to Ice VI. *Nat. Commun.* **2021**, *12* (1), 1128.

(17) Yamane, R.; Komatsu, K.; Gouchi, J.; Uwatoko, Y.; Machida, S.; Hattori, T.; Ito, H.; Kagi, H. Experimental Evidence for the Existence of a Second Partially-Ordered Phase of Ice VI. *Nat. Commun.* **2021**, *12* (1), 1129.

(18) Larson, A. C.; Dreele, R. B. Von. GSAS Manual. **2004**, *748*.

(19) Toby, B. H. EXPGUI, a Graphical User Interface for GSAS. *J. Appl. Crystallogr.* **2001**, *34* (2), 210–213.

(20) Kamb, B.; Prakash, A.; Knobler, C. Structure of Ice. V. *Acta Crystallogr.* **1967**, *22* (5), 706–715.
